# Screening for coronary artery disease among cancer survivors: rationale and design of the REDEEM-CAD study

**DOI:** 10.1186/s40959-025-00308-7

**Published:** 2025-02-04

**Authors:** Cheng Hwee Soh, Joel Smith, Shristy Shrestha, Mark Nolan, Joshua Wong, Nitesh Nerlekar, Thomas H. Marwick

**Affiliations:** 1 Imaging Research Laboratory, Baker Heart and Diabetes Institute, Melbourne, Australia; 2 Baker Department of Cardiometabolic Health, University of Melbourne, Melbourne, VIC Australia; 3 Pre-Clinical Disease and Prevention, Baker Heart and Diabetes Institute, Melbourne, VIC Australia; 4 Department of Cardiology, Peter MacCallum Cancer Centre, Parkville, Melbourne, Australia; 5 Monash University, School of Public Health and Preventive Medicine, Melbourne, VIC Australia

**Keywords:** Coronary artery disease, Cardio-oncology, Coronary angiography

## Abstract

**Background:**

Cancer survivors are reported to be at a heightened risk of coronary artery disease (CAD) due to shared risk factors, potentially cardiotoxic cancer treatments and premature aging in survivors. Early identification of those who are at greater risk, followed by protective treatment, can prevent CAD progression. However, to date there was a relative paucity of prospective data to optimally guide management of atherosclerotic coronary risk among cancer survivors.

**Methods:**

The REDEEM-CAD (Risk-guidEd DisEasE Management plan to prevent CAD in patients with previous cancer) study is a prospective cohort study conducted in Victoria and Tasmania, Australia aiming to evaluate the efficacy of a comprehensive CAD screening strategy. Cancer survivors aged *≥* 40 years with cancer treatment ≥ 5 years prior are eligible for the study. Consented participants will be stratified into low, intermediate or high risk of major atherosclerotic adverse events based on clinical assessment and biochemistry tests. Subsequently, those within the intermediate risk will be referred for coronary artery calcium (CAC) scoring, with computed tomography coronary angiography (CTCA) completed where CAC > 0 and < 400. Participants with high risk or CAC > 400 will be informed about strategies (including lipid-lowering therapy) to manage asymptomatic CAD. Those with low clinical risk or CAC = 0 will conclude their participation while those with CTCA imaged at baseline will be referred for a follow-up CTCA 2-year post-baseline. The primary endpoint is to identify the prevalence of CAD, identified via CAC scoring, among cancer survivors classified as intermediate risk. Secondary endpoint includes the absolute change in total coronary plaque volume over 24 months among those imaged at baseline and follow-up.

**Summary:**

The REDEEM-CAD study will be the first study to systematically evaluate risk of CAD in cancer survivors, and subsequent responsiveness to coronary risk reduction. This will offer valuable insights into the efficacy of the CAD screening strategies among cancer survivors and the impact of treatment on managing plaque progression.

**Trial registration:**

NCT05366153.

## Rationale

Recent advancements in cancer therapies have markedly improved survival rates, leading to a significant increase in the number of cancer survivors. In the United States alone, the population of cancer survivors is estimated to be approximately 18 million as of January 2022, with projections suggesting this figure will continue to rise [1]. While a number of therapies have been pivotal in reducing cancer-related mortality, several have adverse impacts on cardiovascular health [2]. Indeed, the cardiotoxic potential of cancer therapies spans all major treatment modalities to varying degree chemotherapy, radiotherapy, hormone replacement therapy, and immune checkpoint inhibitors [3–5]. Chest radiotherapy has been associated with a significantly increased risk of coronary artery disease (CAD) due to radiation-induced endothelial damage and accelerated atherosclerosis [6]. Epidemiological data indicate that patients with early-stage breast cancer are more likely to succumb to heart disease than to the malignancy itself, highlighting the dual burden of cancer and cardiovascular disease (CVD) as major global health challenges [7]. The 5-year incidence of CAD among treated patients ranges from 0.8 to 2%, encompassing both fatal and non-fatal events [3]. This data underscores the critical need for comprehensive CVD assessment and management in cancer survivors.

Traditional approaches to managing cardiotoxicity have predominantly focused on preventing or mitigating the acute adverse effects that arise during cancer treatment [8]. However, there is a growing recognition that the cardiotoxic effects of chemotherapy and radiotherapy can be potentiated by the presence of additional cardiovascular risk factors, such as obesity, diabetes, and hypertension, which tend to accumulate over time [9]. Consequently, a more proactive approach is warranted, particularly in the elderly cancer survivor population, who are at heightened risk for CAD. Screening for subclinical CAD in this demographic may permit early identification and implementation of atheroprotective strategies to prevent CAD progression. These strategies to manage subclinical CAD should encompass both pharmacological and lifestyle interventions [10]. In particular, the use of statins has been shown to reduce the incidence of major cardiovascular events in high-risk populations, including cancer survivors, by stabilising atherosclerotic plaques, reducing inflammation and ultimately mortality risk [11, 12]. Evidence supports the efficacy of a risk-guided management approach, wherein high risk cancer survivors are provided with targeted cardioprotective treatments, while those at lower risk are spared unnecessary interventions. While current risk-stratified approaches optimize both patient outcomes and healthcare resources, there remains a critical gap in the management of intermediate-risk patients who may exhibit early features of CAD but frequently go untreated. The REDEEM-CAD study addresses this gap through a prospective observational study of a comprehensive screening strategy combining conventional risk stratification with coronary artery calcium (CAC) scoring to guide management. The ultimate goal is to improve long-term cardiovascular outcomes in this population. By providing evidence-based criteria for CAD management, this study has the potential to inform and modify current clinical guidelines, ensuring that cancer survivors receive more targeted and effective cardiovascular care.

## Study design and objectives

### Study design

**Fig. 1:**
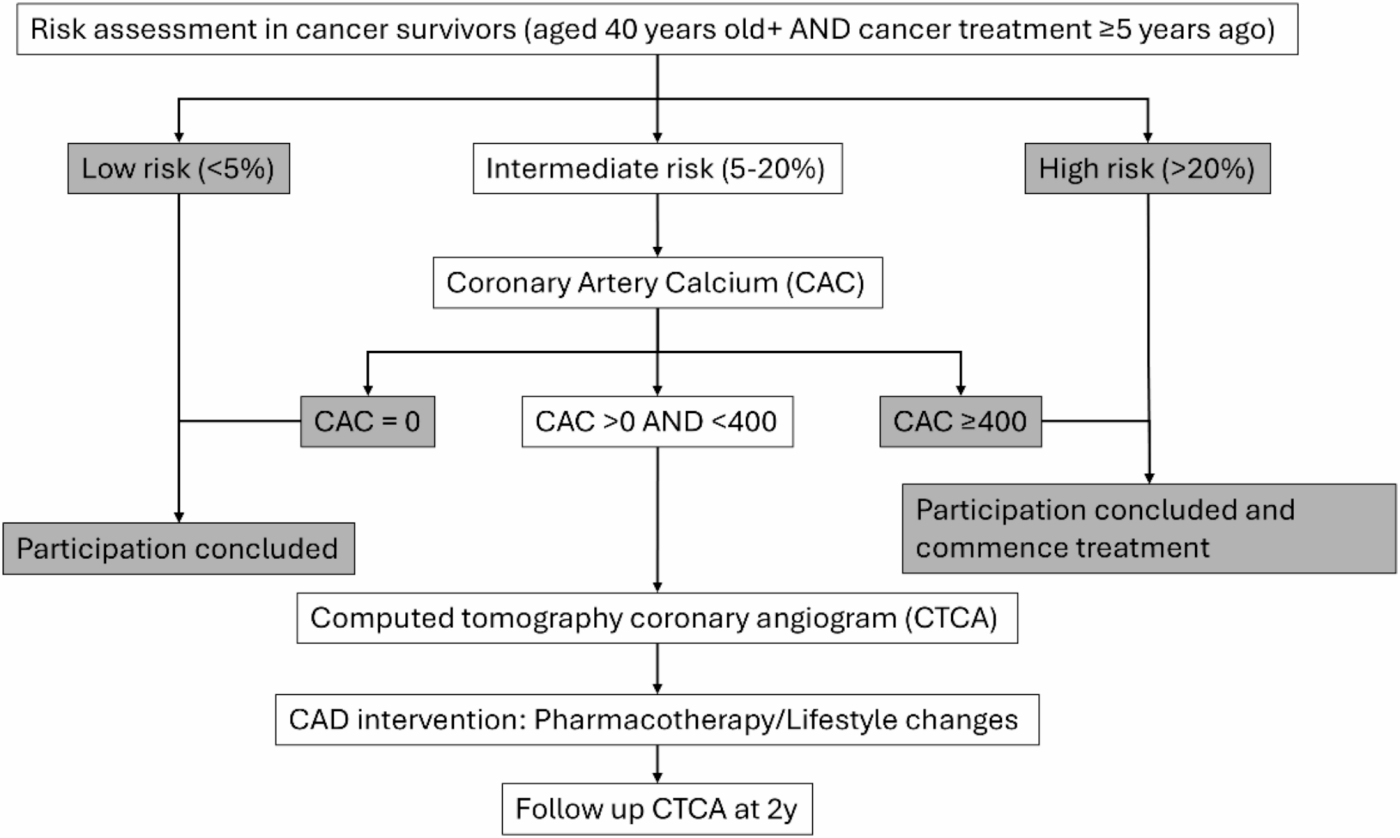
Study design

“*Risk-guidEd DisEase managEMent plan to prevent CAD in patients treated with previous cancer”* (REDEEM-CAD: NCT05366153) is a prospective multicentre study of assessing CAD risk in cancer survivors. This study implements a unique screening/management plan (SMP), which includes 2 components: (i) a novel clinical and imaging-based screening algorithm to select those most likely to develop CAD; and (ii) a clinical review to ensure optimal risk factor control for atheroprotection. The full study design is shown in Fig. 1.

### Support

REDEEM-CAD is supported by an Investigator Grant from the National Health and Medical Research Council of Australia (NHMRC). The authors are responsible for the design and conduct of the study, data analyses, and drafting of the final study report.

### Patient selection

Cancer survivors aged *≥* 40 years, with a treatment history of chemotherapy, radiotherapy, hormone replacement therapy or immune checkpoint inhibitors ≥ 5 years previously are eligible for the study. Exclusion criteria include (i) prior cardiac imaging showing presence of coronary artery disease; (ii) prior myocardial infarction, percutaneous coronary intervention or treatment for stable or unstable angina; (iii) inability to acquire interpretable computed tomography (CT) images; (iv) contraindications/intolerance to, or, already on statin therapy; (v) life expectancy < 12 months or any other medical condition, including pregnancy, that results in the belief that it is not appropriate for the patient to participate in this study; or (vi) inability to provide informed consent.

### Baseline clinical evaluation

Baseline data are collected using validated and standardised methods comprising self-administered questionnaires and clinical assessments. These include age, sex, cancer type and treatment history, ethnicity, employment and income status, alcohol consumption, smoking status, quality of life (EQ-5D-5 L [13] and AQoL-4D [14]), depression status (Patient Health Questionnaires-9 [15], International Physical Activity Questionnaire (IPAQ) [16], Duke Activity Status Index (DASI) [17], height, weight, waist and hip circumference, blood pressure and heart rate, 6-minute walking test [18], medical and medication history, Charlson Comorbidity Index (CCI) [19], electrocardiogram (ECG) and biochemistry tests including fasting glucose level, cholesterol level (total cholesterol, high-density lipoprotein, low-density lipoprotein, triglyceride), and estimated glomerular filtration rate (eGFR).

### Coronary risk assessment

Participants’ risk of major atherosclerotic adverse events are identified based on the Pooled Cohort Equation (PCE) risk score [20] after the completion of baseline assessment and pathology tests. Subsequently, participants are stratified to low (< 5%), intermediate (5–20%) and high (> 20%) 10-year risk of major atherosclerotic adverse events. Participants stratified in groups of low or high risk will conclude their participation in the REDEEM-CAD study as the optimal treatment path for these subgroups is already well-established. Participants at a high risk of cardiovascular events in the next 10-years will be provided guidance and referral to assist in reducing their long-term risk.

Participants with intermediate risk are then referred for CAC scoring [21]. This will be performed on minimum 128-slice CT machines equipped with radiation dose reduction systems and appropriate reconstruction algorithms. Patients will undergo non-enhanced prospective ECG-gated scanning from the angle of carina to the cardiac apex. CAC scoring will be calculated by the Agatston method [22] with scan parameters at a tube voltage of 120 kVp and slice thickness 3 mm. The estimated radiation dose for calcium score is approximately 1mSv (dose length product 90mGy cm). Participants with CAC of 0 will be reclassified as low risk [23] and will not proceed with CTCA. Likewise, participants with CAC ≥ 400 will be reclassified as high risk and will not proceed with CTCA. Participants and their general practitioners (GPs) will be informed of the high risk, with pharmacotherapies recommended to manage the clinical risk.

Those with subclinical CAD (CAC score between 0 and 400) will then undergo same-day CTCA to identify plaque phenotypes and quantify plaque volume. Excellent image quality is essential for plaque volume measurement. Acquisitions are not performed at heart rates > 60 beats per minute. Participants are prepared with oral metoprolol premedication (50 mg in divided doses), and heart rate will be measured on arrival. Additional oral metoprolol or ivabradine may be used to aim for acquisition heart rate of < 60 bpm. Nitroglycerin will be administered ~ 1 min prior to contrast injection. A bolus of 100% lohexal was administered at 6mL/second followed by a 50mL normal saline chaser. Scanning is manually triggered when peak contrast enhancement is observed in the left ventricle, with no enhancement observed in the right ventricle. Tube current will be determined by automatic exposure control on the basis of X-ray attenuation in the initial scout images and the reconstruction kernel. Tube potential will be manually altered by the radiographer, with a default of 100kVp (range 80-120kVp) based on body habitus to minimize radiation dose. Scans will be prospective electrographic triggering, using 70–85% of the phase window with necessary widening on an individualized basis. The estimated radiation dose for full CTCA varies according to participants’ size and heart rate, but is routinely ~ 2 mSv [24]. 

### CAD intervention

All participants identified as high risk or with subclinical CAD (0 < CAC < 400) will be provided with the following guidance:

- *Optimisation of pharmacotherapy*, comprising optimal blood pressure control (early morning target < 140/80mmHg) and provision of lipid lowering therapy with statin therapy, provided by participants’ GPs. Participants are recommended to be treated with atorvastatin 40 mg per day. Intolerance (e.g. myalgia) at this dose is expected in < 5%. In the event of intolerance, the dose will be reduced to 20 mg and if still unsuccessful the medication would be switched to rosuvastatin. Liver function test (LFT) monitoring is recommended prior to and at 12 weeks following both the initiation of therapy and any elevation of dose up to 40 mg per day, and semi-annually thereafter. In the presence of a persistent increase in alanine transaminase (ALT) or aspartate transferase (AST) of > 3 times upper limit of normal, reduction of dose is performed and if still unresolved, drug is withdrawn. If all steps are unsuccessful, the drug will be stopped and the participant will be retained in the trial with follow-up on grounds of intention to treat.
- *Nurse-based coaching*, based on information about risk factor targets, the benefits of changing behaviours, and education about the importance of medication adherence. Behavioural counselling will help develop self-care and management plans. The follow-up process will provide monitoring progress of risk factor management, daily activity, and medication adherence.

### Follow-up

Participants identified as low or high CAD risk based on the PCE risk score, as well as those in intermediate risk with a CAC of 0, will not be followed up. Participants with CAC > 0 and completed CTCA will be followed-up at 12-month post coronary CT appointment. Participants’ intervention plan, determined by participants and their GPs, will be obtained via telehealth.

At 24-month post coronary CT appointment, participants will be referred for a follow-up CT coronary angiography scan. Data including participants’ new disease diagnoses and intervention plans will be evaluated again via in-person clinic appointment. Participants will also be referred for fasting biochemistry tests as per baseline visit.

### Endpoints

The primary endpoint of this study is to determine the prevalence of CAD (evidenced by CAC > 0) among cancer survivors who are classified as intermediate risk based on conventional risk assessment tool (PCE risk score).

Secondary endpoints include the absolute change in total coronary plaque volume over 24 months post CAC, assessed by an independent core laboratory. Additionally, different phenotypes of plaque, including high-risk plaque, non-calcified plaque and calcified plaque, will also be evaluated.

Baseline and follow-up plaque volume studies will be blindly compared in the core laboratory using a workstation for 3-dimensional image analysis. CT plaque analysis software (Medis, The Netherlands) will be used to quantify plaque volume using volume rendering and curved multiplanar reformats are used to evaluate the coronary vessels. Vessels ≥ 2 mm in diameter will be assessed for the presence of plaque. Images undergo semi-automated image extraction using proprietary software. After identifying the plaque area, an automated plaque detection tool will be used to quantify plaque volume on both baseline and follow-up scans [25, 26]. The automated software detects outer vessel wall, lumen and the intervening plaque. However, in each case, the borders of the vessel wall and lumen will be adjusted manually to optimize and confirm software estimates. In cases of mixed plaque phenotype, the borders will also be manually adjusted to correctly separate calcified from noncalcified components. The software then provides volume of both components of mixed plaques. Low-attenuation plaque is identified as plaque with attenuation < 30 HU, as previously published [27]. Leaman score will be calculated at baseline and 24-month follow-up by adding the series of weighted scores calculated for each coronary segment by measuring degree of stenosis, plaque composition and segment location. Our core lab is supervised by an investigator who has completed Level 3 training in CT and has > 10 years of experience in CT research. Baseline and follow-up scans will be analyzed independently by two board-certified cardiologists who have completed CT training and undertaking a consensus process with the core lab supervisor. There is an over-read process for 10% of studies. To quantify inter-reader variability, we will calculate the intraclass correlation coefficient (ICC) for continuous variables and Cohen’s kappa for categorical variables between the two readers on a subset of 30 randomly selected cases.

### Safety

Safety evaluations are performed by recording adverse events, serious adverse events, and by monitoring laboratory parameters, physical examinations, ECGs and vital signs. The following events will be considered: (i) sudden death; (ii) cardiac death; (iii) hospitalisation with angina; (iv) serious arrhythmias requiring treatment; and (v) conduction disturbance requiring a permanent pacemaker implantation. Adverse events arising from CT scanning (e.g. iodinated contrast reaction) will be extracted from the radiology report. Adverse and serious adverse events are gathered, and the study is overseen by an independent data and safety monitoring board.

### Data collection and management

Standardized data acquisition is obtained using case report forms. Data capture, analyses, and archiving are coordinated through a secure web-based database and electronic capture of paper-based case report forms. Assessments are undertaken at a series of mandatory time points with a window of ± 1 month of the scheduled visit to permit flexible scheduling (Table 1). Independent study monitors will verify 5% of study data against source documents.

**Table 1:** Study procedures and timeline for data collection

Study procedures	Baseline	12 m	24 m
Informed consent	x		
Baseline assessment	x		
• Cancer type and treatment • Medical history • Medication • CCI • Height and weight • Waist and hip circumference • 6-minute walking test • ECG • Blood pressure and heart rate			
Self-report questionnaires	x		
• Ethnicity and language • Employment and income status • Alcohol consumption • Smoking status • Patient Health Questionnaires-9 • IPAQ • AQoL-4D • EQ-5d-5 L • DASI			
Clinical review		x	x
• New diagnoses • Medication • Treatment plan • Adverse events • Clinical outcomes			
Pathology results	x		x
PCE risk assessment	x		
CT-imaging	CAC/CTCA in intermediate risk		CTCA in participants imaged at baseline

CCI: Charlson comorbidity index; ECG: electrocardiogram; IPAQ: International Physical Activity Questionnaire; AQoL-4D: Basic Assessment of Quality of Life; DASI: Duke Activity Status Index; PCE: Pooled cohort equation; CT: Computed tomography; CAC: Coronary artery calcium; CTCA: Computed tomography coronary angiogram

### Statistical analyses

All data will be pooled and summarised with respect to demographic and baseline characteristics. Participants are then stratified based on their CAD risk (determined according to the PCE risk score). Exploratory data analyses will be performed using descriptive statistics, with categorical data being reported as number (percentage) whereas continuous variables as mean ± standard deviation (if parametric) or median [interquartile range] (if non-parametric).

Within the participants identified as intermediate risk by PCE, the proportion of participants with CAC > 0 will be determined, and the distribution of CAC score will be reported. Those who completed CTCA will then be included in the subsequent analysis identifying the absolute change in total coronary plaque volume, as well as the phenotypes of high-risk plaque, non-calcified plaque and calcified plaque, over 24-month post CAC. Participants will then be stratified based on statin use at 24-month post CAC and the differences in the absolute change in total plaque volume will be identified via t-test. The prevalence of critical CAD, defined as coronary stenosis > 70% identified via CTCA, will also be evaluated. The efficacy of this CAD risk evaluation will be compared with the broad community in two existing studies – (i) the CAUGHT-CAD trial [28] and (ii) the EDCAD-PMS trial (NCT04604353, ongoing).

Missing data will be explored and the nature of missing data will be identified. Multiple imputation by chain equation (MICE) will be applied to deal with data missing at random (MAR). Sensitivity analyses will be explored if missing data were identified as missing not at random (MNAR). These analyses will be on grounds of intention-to-treat.

Subgroup analyses, stratified based on age range, sex and cancer types, will also be conducted. A *p*-value of < 0.05 will be considered as statistically significance. All statistical analyses will be conducted using R (R Foundation for statistical computing).

### Power calculations

Sample size calculations were based on our previous study in a non-cancer cohort (CAUGHT-CAD trial), which demonstrated that 48% of participants classified as intermediate risk had CAC > 0 and warranting preventative therapy. Given the increased cardiovascular risk associated with cancer survivorship, we hypothesized a 10% higher prevalence of significant CAD in our study population compared to the general population. Using a two-sided alpha of 0.05 and power of 80%, we calculated that 839 participants would be required to detect this anticipated difference in CAD prevalence. This sample size accounts for potential attrition and provides sufficient statistical power to address our primary endpoint of CAD prevalence in intermediate-risk cancer survivors.

## Preliminary data

The REDEEM-CAD study initiated on October 2023. Table 2 demonstrates participants’ sociodemographic and clinical characteristics at baseline. In summary, participants had a median age of 64 [54–70] years and the majority of them were survivors from breast cancer (*n* = 49). The proportion of survivors who went through chemotherapy and radiotherapy were 66% and 86%, respectively. The overall comorbidity burden was mild to moderate (median CCI: 2 [0–3]), with dyslipidaemia being the most prevalent comorbidity among the survivors (*n* = 21). The total cholesterol, high-density lipoprotein (HDL) and low-density lipoprotein (LDL) levels were 5.4 [4.6–5.9], 1.6 [1.4-2.0] and 3.1 [2.4–3.7], respectively. Based on the clinical assessment and pathology results, survivors showed median PCE risk score of 6.0% [2.1–11.8], with the distribution of low-, intermediate- and high-risk being *n* = 47, *n* = 41 and *n* = 12, respectively. The median CAC score for the survivors within intermediate risk were 14 [0–45].

**Table 2:** Baseline characteristics of the consented participants

	Total (*n* = 100)	Low PCE risk (*n* = 47)	Intermediate PCE risk (*n* = 41)	High PCE risk (*n* = 12)
Age, years	64 [54–70]	55 [45–60]	66 [64–71]	74 [72–77]
Male, n	19	3 (6.4%)	9 (22.0%)	7 (58.3%)
Body mass index, kg/m^2^	26.3 [24.1–30.7]	25.6 [23.9–27.7]	26.7 [24.3–31.9]	27.9 [25.2–30.6]
Cancer type, n				
Breast cancer	49	23 (48.9%)	24 (58.5%)	2 (16.7%)
Lymphoma	39	19 (40.45%)	12 (29.3%)	8 (66.7%)
Leukaemia	2	1 (2.1%)	1 (2.4%)	0
Other cancer type	9	4 (8.5%)	4 (9.8%)	1 (8.3%)
Smoking status, n				
Current smoker	2	1 (2.1%)	1 (2.4%)	0
Former smoker	34	17 (36.2%)	13 (31.7%)	4 (33.3%)
Never smoke	64	29 (61.7%)	27 (65.9%)	8 (66.7%)
Blood pressure, mmHg				
Systolic blood pressure	130 [119–144]	121 [111–132]	135 [128–149]	141 [127–160]
Diastolic blood pressure	80 [72–89]	79 [71–86]	81 [72–90]	81 [71–88]
Heart rate, bpm	76 [69–81]	76 [70–80]	76 [69–81]	71 [64–88]
Cancer treatment, n				
Chemotherapy	66	35 (74.5%)	22 (53.7%)	9 (75%)
Radiotherapy	86	44 (93.6%)	34 (82.9%)	8 (66.7%)
Comorbidities, n (%)				
Atrial fibrillation	2	0	1 (2.4%)	1 (8.3%)
Arrhythmia	7	5 (10.6%)	1 (2.4%)	1 (8.3%)
Cerebrovascular disease	2	1 (2.1%)	1 (2.4%)	0
Chronic kidney disease	1	0	0	1 (8.3%)
Deep vein thrombosis	3	1 (2.1%)	0	2 (16.7%)
Diabetes	5	1 (2.1%)	1 (2.4%)	3 (25%)
Dyslipidaemia	21	9 (19.1%)	7 (17.1%)	5 (41.7%)
Psychiatric disorder	4	2 (4.3%)	2 (4.9%)	0
Respiratory disease	2	1 (2.1%)	1 (2.4%)	0
Charlson Comorbidity Index, score	2 [0–3]	2 [0–4]	2 [0–2]	0 [0–2]
Duke Activity Status Index, score	45 [37–58]	51 [38–58]	45 [39–51]	35 [28–41]
6-minute walking distance, m	580 [520–629]	582 [535–640]	589 [521–620]	494 [426–603]
Completion of expected distance, %	112 [101–124]	107 [98–115]	121 [109–131]	104 [91–131]
Total cholesterol, mmol/L	5.4 [4.6–5.9]	5.0 [4.2–5.8]	5.6 [5.0-6.1]	5.9 [4.6–6.4]
HDL cholesterol, mmol/L	1.6 [1.4-2.0]	1.7 [1.5-2.0]	1.5 [1.3–1.9]	1.5 [1.2–2.1]
LDL cholesterol, mmol/L	3.1 [2.4–3.7]	2.9 [2.2–3.4]	3.5 [2.7–3.8]	3.5 [2.5-4.0]
Triglyceride, mmol/L	0.9 [0.8–1.3]	0.9 [0.7–1.1]	0.9 [0.8–1.5]	1.3 [0.8–2.7]
PCE risk score, %	6.0 [2.1–11.8]	2.0 [0.7–3.4]	10.2 [6.9–13.8]	24.9 [21.0-36.6]

HDL: High density lipoprotein; LDL: low density lipoprotein; PCE: Pooled cohort equation

## Context

### Disentangling the association between cancer and CAD

Cancer and CAD share several underlying mechanisms, primarily involving inflammation, oxidative stress, and endothelial dysfunction [29]. Both conditions are associated with heightened inflammatory responses and increased oxidative stress, which contribute to endothelial injury and atherogenesis. Chronic inflammation, commonly seen in cancer, leads to DNA damage and tumor progression, which parallels the inflammatory processes driving atherosclerosis. Additionally, cancer therapies, including chemotherapy and radiotherapy, exacerbate CAD risk by causing endothelial damage, further accelerating atherosclerosis and increasing cardiovascular morbidity in survivors [30]. 

The association between cancer and CAD is also influenced by shared traditional cardiovascular risk factors that are often prevalent among cancer survivors, such as obesity, smoking, and diabetes [31, 32]. These risk factors not only contribute to the development of CAD but also interact with cancer-related pathways to exacerbate cardiovascular risk. For instance, obesity-associated inflammation and insulin resistance [33], smoking-induced endothelial dysfunction [34], and diabetes-related oxidative stress [35] all interplay with cancer’s inflammatory and oxidative mechanisms to heighten CAD risk. Understanding these interconnected pathways is crucial for developing targeted strategies to prevent and manage CAD in cancer survivors.

### Decision on risk stratification approach prior CTCA

While CAD is commonly identified among cancer survivors [36], the traditional approach to cardiovascular risk assessment, mostly developed for the general population, may not adequately capture the complex risk profile of cancer survivors. Current risk prediction tools, such as the PCE and AUCVD risk score, were not specifically validated in cancer survivors and may underestimate their true cardiovascular risk [37]. Previous retrospective cohort study showed that the prevalences of CVDs are higher among cancer survivors than non-cancer controls with similar CAC scores [38]. This creates a need for targeted screening strategies that consider both traditional cardiovascular risk factors and the effectiveness of an additional testing (CAC scoring) to guide CAD management.

Furthermore, the optimal timing and method of cardiovascular screening in cancer survivors remain unclear. While some guidelines recommend early screening for specific cancer populations, there is no standardized approach for comprehensive CAD assessment in the broader cancer survivor population. This gap in clinical practice, combined with the increasing life expectancy of cancer survivors, highlights the need for evidence-based screening strategies that can effectively identify those at highest risk for CAD.

Performing CTCA on all participants in the REDEEM-CAD study, rather than using a risk stratification approach, might seem like a straightforward solution for assessing CAD in cancer survivors. However, several compelling reasons support the decision to implement risk stratification before conducting CTCA.

1. *Resource optimization and cost-effectiveness.* CTCA is a valuable diagnostic tool for CAD evaluation, providing detailed imaging of coronary arteries and allowing for the assessment of plaque characteristics [39]. However, it is resource-intensive in terms of financial costs as performing CTCA on all cancer survivors would lead to substantial increases in healthcare cost. Given that more than half of the participants in the EDCAD-PMS trial undergoing CAC had a score of 0, conducting CTCA procedures on all survivors could be unnecessary. By implementing a risk stratification process, the study effectively triages participants based on their likelihood of having CAD. This targeted approach ensures that CTCA is reserved for those most likely to benefit from it—those with intermediate PCE risk where pharmacotherapy might be unnecessary [40]. This strategy reduces the number of CTCA procedures performed, optimizing resource use and minimizing costs while ensuring that patients with significant risk receive appropriate diagnostic evaluations and corresponding treatment, if applicable.
2. *Reduction in radiation exposure.* CTCA involves exposure to ionizing radiation, which, while relatively low in dose, accumulates over repeated exposures. For cancer survivors, who may have already undergone significant radiation exposure as part of their cancer treatment, additional radiation exposure could pose additional health risks. By using PCE risk scores and CAC to filter participants, the study minimizes unnecessary CTCA procedures and thus limits radiation exposure to those who are more likely to benefit from detailed imaging [29]. This risk-based approach helps to strike a balance between diagnostic accuracy and patient safety, aligning with the principle of minimizing harm while still providing necessary care. The reduction in radiation exposure is particularly important for cancer survivors, who may be more sensitive to the risk from additional radiation due to their previous treatments.

In summary, the decision to employ risk stratification prior to CTCA in the REDEEM-CAD study is driven by considerations of resource optimization, reduction in radiation exposure, and clinical utility. This approach ensures that CTCA is used judiciously and effectively, maximizing benefits for participants while minimizing unnecessary procedures and associated risks.

### Limitations

The key limitation of this study is the challenges in risk stratification accuracy. The initial risk stratification is fully based on PCE, which may underestimate the proportion of cancer survivors who require further screening. While universal scanning would provide more complete data on PCE’s performance in this population, our study design deliberately mirrors real-world clinical practice constraints, where resource limitations necessitate selective screening approaches. This pragmatic approach, though potentially missing some at-risk individuals, aims to establish an implementable framework for cardiovascular risk assessment in cancer survivorship care. Similarly, CAC may not detect all forms for subclinical CAD, particularly in early stages or in cases where plaque is non-calcified. These could potentially limit the validity of the study outputs. No screening test is perfect, and it is possible for a patient to have asymptomatic, severe non-calcified atherosclerotic disease that would be missed on a CAC scoring test and lead to inappropriate delay of treatment. Current evidence suggests that this clinical scenario is very uncommon, with a meta-analysis indicating that prevalence of obstructive non-calcified plaque is 1.1% and we may therefore incorrectly designate approximately 9 patients as low risk in our target sample size [41]. This does not justify the cost and radiation exposure of this strategy. Our methodology in employing CAC as an additional screening tool aligns with current clinical practice guidelines and offers a balance between diagnostic yield and resource utilization in risk stratification. While we do provide a guideline and recommendation to participants and their GPs, the final intervention to manage CAD risk lies at the GPs’ discretion and their discussion with the participants. Thus, the differences in clinical care might result in disparity in the outcome of interest. We plan to address this by conducting sensitivity analyses to adjust for participants’ treatment plan at follow-up.

## Conclusion

The growing recognition of the increased risk of CAD among cancer survivors necessitates a proactive and evidence-based approach to management. The REDEEM-CAD study will test a comprehensive strategy that combines risk stratification, advanced imaging techniques, and tailored interventions to manage and mitigate CAD risk in this vulnerable population. We aim to improve long-term cardiovascular outcomes for cancer survivors, ultimately reducing the dual burden of cancer and coronary artery disease. The results of this study have the potential to inform clinical guidelines and practice, ensuring that cancer survivors receive the most effective care for their cardiovascular health.

## Data Availability

No datasets were generated or analysed during the current study.

## Abbreviations

ALT: Alanine aminotransaminase
AST: Aspartate aminotransferase
CAC: Coronary artery calcium
CAD: Coronary artery disease
CCI: Charlson Comorbidity Index
CT: Computed tomography
CTCA: Computed tomography coronary angiography
ECG: Electrocardiogram
LFT: Liver function test
MAR: Missing at random
MNAR: Missing not at random
MICE: Multiple imputation by chain equation
PCE: Pooled cohort equation
SMP: Screening and management plan
